# Photonic gas sensors exploiting directly the optical properties of hybrid carbon nanotube localized surface plasmon structures

**DOI:** 10.1038/lsa.2016.36

**Published:** 2016-02-26

**Authors:** Thomas Allsop, Raz Arif, Ron Neal, Kyriacos Kalli, Vojtěch Kundrát, Aleksey Rozhin, Phil Culverhouse, David J Webb

**Affiliations:** 1 Aston Institute of Photonic Technologies, School of Engineering and Applied Science, Aston University, Aston Triangle, Birmingham B47ET, UK; 2 Nanoscience Research Group, School of Engineering and Applied Science, Aston University, Aston Triangle, Birmingham B47ET, UK; 3 Physics Department, Faculty of Science, University of Sulaimani, Sulaimani, Iraq-Kurdistan Region; 4 Faculty of Science and Technology, School of Maths, Computing and Robotics, University of Plymouth, Plymouth PL4 8AA, UK; 5 Department of Electrical Engineering, Computer Engineering and Informatics, Cyprus University of Technology, Limassol 3036, Cyprus

**Keywords:** carbon nanotubes, gas sensors, localized surface plasmons, optical sensing

## Abstract

We investigate the modification of the optical properties of carbon nanotubes (CNTs) resulting from a chemical reaction triggered by the presence of a specific compound (gaseous carbon dioxide (CO_2_)) and show this mechanism has important consequences for chemical sensing. CNTs have attracted significant research interest because they can be functionalized for a particular chemical, yielding a specific physical response which suggests many potential applications in the fields of nanotechnology and sensing. So far, however, utilizing their optical properties for this purpose has proven to be challenging. We demonstrate the use of localized surface plasmons generated on a nanostructured thin film, resembling a large array of nano-wires, to detect changes in the optical properties of the CNTs. Chemical selectivity is demonstrated using CO_2_ in gaseous form at room temperature. The demonstrated methodology results additionally in a new, electrically passive, optical sensing configuration that opens up the possibilities of using CNTs as sensors in hazardous/explosive environments.

## Introduction

Carbon nanotubes^1^ (CNTs) came to prominence in the 1990s and have since attracted significant research effort because of their interesting electrical, optical, mechanical, and thermal characteristics^2,3^ which have suggested many potential applications in the fields of nanotechnology, environmental sensing, and biochemistry^4^. There has been considerable success in functionalizing CNTs to provide a specific response to various chemicals^5,6,7^, enabling the selective detection of very low concentrations of gases^8,9,10^ and vapours^11,12^ with good repeatability. To date, selective gas sensing has been carried out by monitoring the changes in the electrical properties of the CNTs; to do so optically has posed significant challenges.

Recently, we have developed a plasmonic sensing platform based upon localized surface plasmons (LSPs). Surface plasmon resonance is an important optical phenomenon that involves a resonant transfer of incident propagating light to a surface plasmon mode^13,14^, which takes the form of collective electron oscillations at the interface between a dielectric and metal^14^. In our approach, the plasmons are generated by a nanostructured thin film that resembles an array of nano-wires that are capable of detecting ultra-small changes in the refractive indices of surrounding liquids or gases^13^. This optical sensing platform working in conjunction with immobilized-specific chemical receptors has previously been shown to have the ability to detect sub nano-molar concentrations of chemicals in a small volume of solution^15^.

Using this sensing platform in conjunction with immobilized CNTs on its surface, we are able to measure for the first time the changes in optical properties of the CNTs caused by the specific interplay with a given chemical species, beyond the changes usually associated with bulk modification of the refractive index^16^. In particular, we show that combining this platform with CNTs enables a specific response to CO_2_ to be observed just by monitoring the optical properties of the CNTs.

Recent experimental studies have shown that CNTs have an affinity with carbon dioxide (CO_2_), which causes an increasing electron density resulting in hole depletion that effects the electrical properties of the CNTs^17,18,19^. Here we are the first to show that CO_2_ induces chemically driven changes in the optical properties of the CNTs and furthermore demonstrate that this physical phenomenon can be exploited for specific chemical sensing applications. The chemical selectivity to the CO_2_ molecule is proven by a comparison with the results for other gaseous molecules at normal atmospheric conditions. The alkane gases methane, ethane, propane, and butane were used where methane and ethane are of similar size to CO_2_. It is important to stress that whilst the monitoring of CO_2_ is an important application in its own right, the approach demonstrated here is far more generic; it is now well established that CNTs can be functionalized to provide specific responses to many other chemical species^4,5,6^. Moreover, it is now well established that there are significant advantages to having all-optical sensing technology; for example, in the field of gas sensing in explosive environments a key feature is the removal of any electrical spark hazard.

## Materials and methods

### LSP sensing platform fabrication.

First, a standard single-mode optical fiber was mechanically lapped down to 10 μm from the fiber central axis producing a D-shaped fiber with an approximate 5 μm width between the core/cladding interface and the flat of the D. This separation distance is large enough to minimize the evanescent field strength at the flat of the lapped fiber surface and to stop the coated flat of the D-shaped acting as a “mode sink” which would affect the overall optical dynamic range of the sensor.

Second, using an RF sputtering machine (Nordico 6 inch RF/DC 3 target excitation machine, Nordiko 6, Nordiko Technical Services Limited, Havant, Hampshire, UK), a series of coatings was deposited upon the flat of the lapped fiber. These coatings consisted of layers of germanium (48 nm), silicon dioxide (48 nm), and platinum (36 nm), the reasoning for using the specific materials and thicknesses is given below.

Third, the coated fiber was exposed to a 244 nm ultraviolet (UV) light interference pattern produced by a uniform phase mask with period 1.018 μm (a standard fiber-grating phase mask) illuminated by an argon ion laser (Sabre Fred Coherent Inc laser, Coherent Inc, Santa Clara, California, USA). At the point of inscription the laser delivered 110 mW of power and the laser beam was scanned at 0.05 mm s^−1^ over the coated fiber for multi-exposure, typically seven times. This produced a surface relief structure which has dominant spatial periods of ∼0.5 and ∼1 μm, described fully in a previous publication^14^. The spectral features of the fiber devices are monitored using a linearly polarized, broadband light source.

The surface relief structure induces a strain field that causes an asymmetric radial index variation across the cross-section of the D-shaped fiber, which can be envisaged as a radially symmetric index profile in a curvilinear waveguide (by the conformal mapping technique^20^) and helps to efficiently couple light to the surface plasmons. The rationale for using these materials is in two parts. The first concerns the optical constants of the materials and how their dispersion relationships allow coupling to surface plasmons at a metal-dielectric or semiconductor-dielectric interface; both Ge and Pt exhibit this behavior. Second, Ge and SiO_2_ layers are used due to the fact that it is known from studies of grating formation^21^ that when exposed to UV light, Ge/GeO produces photo-bleaching and compaction of the material, thus producing a surface corrugation on the multi-layered structure.

**Figure 1:**
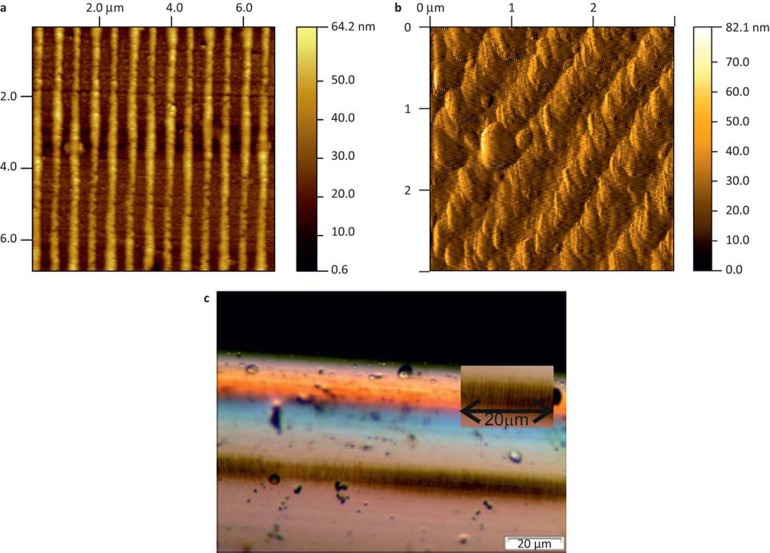
Images and topological data of the post UV-laser processed device. (**a**) and (**b**) are AFM images showing respectively the linear structures created and the finer detailed structure of the surface topology. (**c**) is a visible microscope image with a magnified insert.

The surface topology is shown in Figure 1. Measurements of the surface structure, using an atomic force microscope (AFM) and X-ray photoelectron spectroscopy indicate that the surface consists of an array of platinum nano-wires (Figure 1a), typically 36 nm in radius and 20 μm in length, supported by the silicon dioxide thin film on a thin substrate of germanium^22^. The nano-wires are perpendicular to the longitudinal axis of the D-shaped fiber. Figure 1b shows the finer structure of the nano-wires, which may be responsible for the large spectral tunability of these LSP devices. The mechanism that creates the surface corrugation seen in Figure 1 is still being investigated, though there is evidence suggesting that the UV irradiance generates the surface topology by the creation of germanium oxides (by a photo-bleaching process) that in turn produces a stress-field that governs the growth of the structures^22^. Following the surface structuring and some initial characterization, CNTs were attached to the surface as described below.

**Figure 2:**
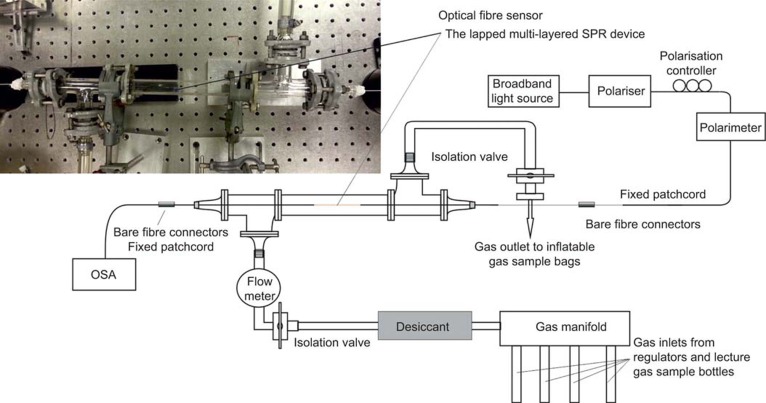
Scheme of gas sensing apparatus and picture of the gas chamber.

It is known that the polarization properties of the illuminating light affect the spectral characteristics of surface plasmons^15^. To ensure that the sensitivity was maximized, the devices were characterized by measuring their spectral dependence as a function of the azimuthal polarization properties of the illuminating light; the results are discussed later. Light from a broadband light source (Agilent 83437A Broadband Light Source, Agilent Technologies Inc, Santa Clara, California, USA), was passed through a polarizer (broadband internal polarizer for polarimeter PAT 9000B) and a polarization controller (manual fiber paddle polarization controller) before illuminating the sample, with the transmission spectrum being monitored using an optical spectrum analyzer (OSA, Model 86140 Agilent range from 600 to 1700 nm with an accuracy of 5 pm). The change in polarization of the illuminating light was monitored with a polarimeter (Tektronix, PAT 9000B, Tektronix UK Ltd. Bracknell, Berkshire, UK) through a polarization maintaining coupler. An investigation was carried out into the spectral dependence of the LSP resonances as a function of the surrounding medium’s refractive index, for both the liquid and gaseous index regimes, using the optical part of the apparatus shown in Figure 2. The sensitivity of the devices at low refractive indices was determined using the alkane gases (methane, ethane, propane, and butane), while higher indices were obtained from certified refractive index (CRI) solutions. In the aqueous regime the fibers were placed in a V-groove and immersed in CRI liquids (Cargille Laboratories, Cargille-Sacher Laboratories, New Jersey, USA) that have a quoted accuracy of ±0.0002. The experiment was carried out both before and after CNTs were adhered to the sensor.

**Figure 3:**
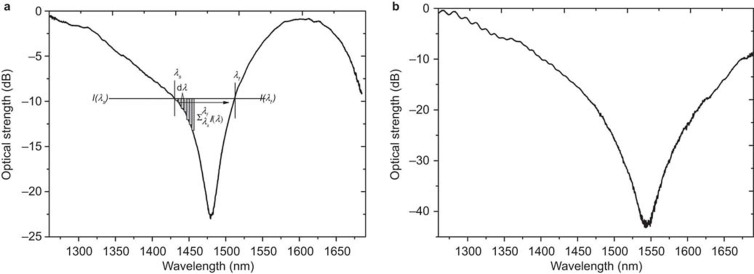
(**a**) shows the spectral transmission feature of the LSP (UV processed with no CNTs) fiber sensor submerged in a solution with a refractive index value of 1.32 and the visualization of determining wavelength shift. (**b**) Shows the spectral transmission feature of the same LSP fiber sensor in **a** but with the coating of CNTs.

Due to the broadness of the spectral transmission features that need to be analyzed (see Figure 3a and 3b for examples) the central wavelength is calculated by the first moment of the power spectrum: the centroid by geometric decomposition^23^. The centroid is given by: 
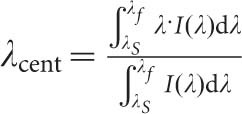
, where *λ*_cent_ is the centroid wavelength over a range of *λ_s_* to *λ_f_* and *I_i_* are the associated amplitude/intensities measured in dBs over the part of the spectrum between the points at −10 dB of the maximum transmission, where the surface plasmon resonance exists. The associated centroid strength value is calculated as the mean value over the same interval range of interest, Figure 3a gives a visual representation of this evaluation procedure on experimental data from an LSP (UV processed with no CNTs) fiber sensor. Figure 3b shows the spectral response of the same LSP fiber sensor with an adhered coating of CNTs.

We used a wet chemistry route for the coating of the LSP sensor with purified single wall CNTs (CoMoCaT CG 200, SouthWest NanoTechnologies Inc., Norman, Oklahoma, USA). First, 0.5 mg of CNTs were dispersed in 10 ml of N-methyl-2-pyrrolidone (NMP) via sonication (20 kHz, 200 V, 1 h, Nanoruptor, Diagenode SA, Liege, Belgium). The use of NMP is conditioned by its efficiency in the direct dispersion of CNTs (hydrophobic material) at concentrations below 0.02 mg ml^−1^ ^24^. Additionally, we added polyvinyl pyrrolidone (PVP) polymer (1 mg ml^−1^) as a dispersion agent in order to achieve higher concentrations of CNTs within the resulting dispersion^25^. In order to achieve a highly uniform dispersion and remove residual CNT bundles, the CNT-PVP-NMP system was centrifuged for 30 min at 10 000 RPM with MLS-50 rotor (Optima MaxXP Benchtop Ultracentrifuge, Beckman Coulter, Brea, California, USA). The surface plasmon resonance fiber device was placed subsequently in the micro-capillary tube filled with the CNT dispersion for a few minutes. Finally, the resulting device was dried in air at atmospheric pressure for 24 h before placing in the gas line.

## Results and discussion

### Spectral sensitivity before adhesion of carbon nanotubes

**Figure 4:**
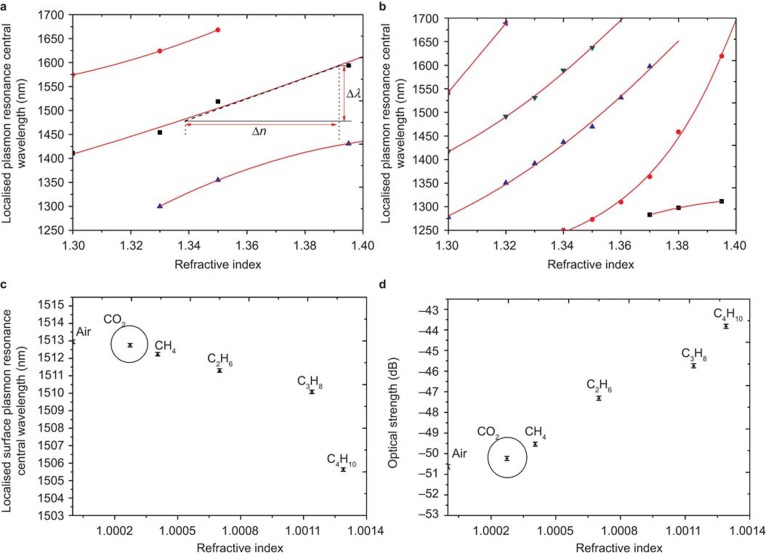
Spectral sensitivities, before the adhesion of the CNT coating, with respect to refractive index in (**a**) the aqueous regime prior to UV laser processing, where conventional surface plasmons are generated from the multi-layered coating of the optical fiber; (**b**) the aqueous regime following UV laser processing, where LSPs are generated. The spectral sensitivity of the LSP sensor in the gaseous index regime following UV laser processing for a resonance at a nominal wavelength of 1510 nm, (**c**) the wavelength sensitivity and (**d**) the associated change in optical strength. All gases are flowed at one atmosphere pressure.

Figure 4 shows the spectral sensitivity prior to UV laser processing (Figure 4a) and the changes that occur following UV processing of the multi-layered coating (Figure 4b). There are several surface plasmon resonances observed at different spectral locations that are dependent upon the azimuthal polarization condition of the illuminating light. The spectral sensitivity to changes in the surrounding refractive index is dramatically increased following UV processing. Prior to UV laser processing, the highest measured index sensitivity was Δ*λ*/Δ*n* ∼ 2070 nm RIU^−1^ (refractive index unit), see Figure 4a, which shows how the spectral sensitivity is estimated, whereas following processing the maximum increases substantially to Δ*λ*/Δ*n* ∼ 10 700 nm RIU^−1^. Both results are measured in the important aqueous index regime (1.36–1.39). Furthermore comparing Figure 4a to 4b, there are more resonances in the transmission spectra. This dramatic change in spectral behavior is expected because the UV processing transforms the conventional surface plasmon device to an LSP device. This transformation can be visualized by considering the surface of the device before UV processing which is a plane uniform surface of gold that interacts with the environment and across which the surface plasmons traverse. Therefore the generated surface plasmons’ physical properties, such as propagation length, resonant condition, and spatial extension from the surface are dependent on gold thickness, roughness, topology, and effective refractive index of the coating with the surrounding environment^15^. This results in a number of resonances in the wavelength range of interest. After UV processing, first the surface topology transforms to a corrugation with a more complex-repeated structured on the apex of the corrugations (see Figure 1a and 1b) composed of materials having different properties (metal, semiconductor, and dielectric). The surface plasmons that now exist on this new structure can only propagate along the individual regions of metal (the apexes) and thus they are confined to these specific regions as LSPs. Furthermore, the shape of the metal regions are important in the spectral behavior and resonance of the LSP^26^. The two different geometries pre- and post-UV processing result in different resonant conditions and numbers of resonances.

In the gaseous regime the wavelength shift and change in optical strength are shown in Figure 4c and 4d, respectively, for a resonance at 1510 nm. This reveals a refractive index sensitivity of Δ*λ*/Δ*n* ∼ −6200 nm RIU^−1^, whereas the change in optical strength reaches Δ*I*/Δ*n* ∼5900 dB RIU^−1^. At this stage of fabrication, if we consider the unfinished fiber device as a sensor then the authors believe that this is the highest reported spectral sensitivity to bulk index changes within the gaseous regime, compared with other fiber optic sensors^27,28,29,30^. Furthermore it is noted that in the lower refractive index regime, there is a blue wavelength shift with increasing refractive index compared with a red wavelength shift in the higher index regime. This behavior is first due to the fact that the gas resonances are different surface plasmon resonances than are shown in Figure 4a and 4b and second the dispersion relationship is an important factor in how the surface plasmons spectrally shift in response to changes in environmental parameters^15^.

Note this class of devices can be tailored for refractive index spectral sensitivity in different refractive index regimes by altering the structure, such as using different thicknesses of gold or silver as the metal overlay or changing the thickness of the other sub layers in the multi-layered coating^14,22,31^ or changing the UV processing conditions^22^.

### Chemical sensing: Specific chemical spectral response of carbon nanotubes

**Figure 5:**
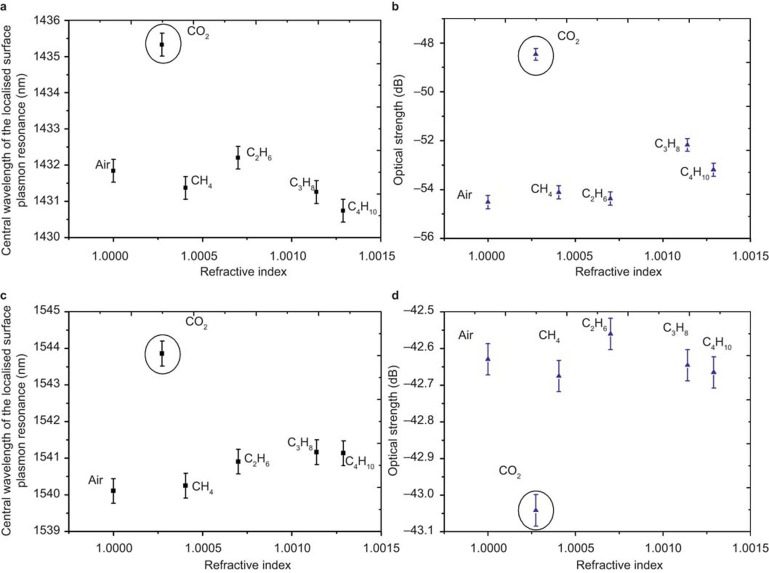
The demonstration of the LSP sensor using resonances at 1430 nm (**a**) and (**b**), and 1540 nm (**c**) and (**d**).

After the CNTs were adhered to the surface of the fiber platform, the resulting device was placed within the gas chamber (Figure 2) and the changes in the CNTs’ optical constants (permittivity, permeability, refractive index, and extinction coefficient) were observed via the sensor’s spectral index sensitivity. These results yielded the specific spectral response to CO_2_, the limit of detection of CO_2_, and the influence of polarization. Comparisons were made with the device prior to coating with CNTs. With the addition of the CNTs, two LSP resonances were observed with central wavelengths at 1540 and 1430 nm with optical strengths (the extinction ratio of the optical power level at the center of the plasmon resonance in the transmission spectrum compared to the power off-resonance) of 41 and 50 dB, respectively; the spectral dependence on the gases is shown in Figure 5.

There are several observations that can be made with regards to Figure 5. First, the sensor registers a large wavelength shift (Δ*λ* ∼3.8 nm) in the presence of CO_2_ compared to the alkane gases. The lack of a similar CO_2-_specific response in Figure 4, obtained from a sensor assembled without CNTs, confirms that the sensitivity to CO_2_ is a direct result of the addition of the CNTs and a reaction to the presence of CO_2_. This demonstrates a large difference between the response to bulk refractive index and the chemically induced changes in the optical properties, caused by the specific interplay between the CNTs and CO_2_^16,32,33^; up till now the only proven chemically selective CNTs sensor have been electrically based^34^.

It is known that other gaseous compounds, such as N_2_^35^, can act as a redox agent^36^ to the CNTs, but this is usually at temperatures in excess of 500°C, suggesting that high temperatures are required to observe a substantial reaction. Furthermore, the experiments done here start with the sensor exposed to a normal earth’s atmosphere that contains a large percentage of N_2_ and nevertheless a large spectral wavelength shift was still observed with the addition of CO_2_. This suggests that for CO_2_ the activation energy for a redox reaction with CNTs is much lower that N_2_, thus at nominal ambient temperatures the CO_2_ reaction dominates over that of N_2_. At high temperatures though, the chemical selectivity may be reduced.

To enable a comparison with other researchers’ results, we note that this behavior yields equivalent spectral index sensitivities in excess of 3 × 10^4^ nm RIU^−1^ and 4.2 × 10^4^ dB RIU^−1^, in a CO_2_ atmosphere approaching a 100% concentration, which leads to an equivalent index resolution of ∼10^−5^ ^37^. The spectral response to bulk refractive index changes prior to the addition of CNTs is 805 nm RIU^−1^ and −13 dB RIU^−1^; approximately one order of magnitude less. This result indicates that the CNT coating is acting as a shield to reduce the overall effect of the change in the bulk index sensitivity to the surrounding material. Finally, Figure 5c shows an opposite wavelength shift associated with an increase in the surrounding medium’s index in the gas index regime compared with Figure 4c. The addition of CNTs, and their supporting polymer, will increase the effective refractive index around the sensing structure and perhaps more importantly change the topology that would in turn change the overall dispersion relationship of the LSPs that governs the spectral resonance shift and sensitivity.

**Figure 6:**
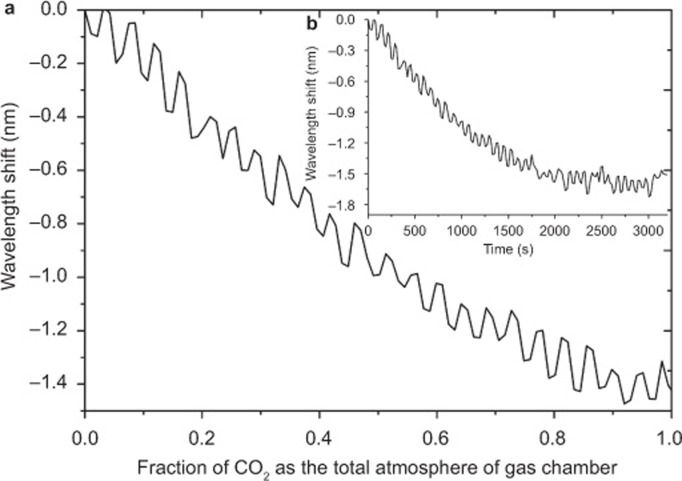
Typical spectral behavior of the fiber sensor with an LSP wavelength resonance at 1390 nm, (**a**) as a function of the fraction of CO_2_ in the surrounding atmosphere (**b**) showing the spectral response of the sensor with respect to the time taken for the experiment to be completed.

As a final test, the CNT-based device was monitored in order to observe wavelength shifts in response to the continuous flow of CO_2_, changing the environment from standard atmospheric conditions to a saturated atmosphere of CO_2_ with an inlet flow rate of 0.5 liters min^−1^. Typical results are shown in Figure 6a and 6b from which we can also extract a response time for the chemically-induced optical changes. The superimposed, small slow sinusoidal variation in Figure 6 has a major frequency component of 0.013 Hz and represents a repeatable systematic error with a low frequency component which may be attributable to a small mechanical vibration of the suspended optical fiber caused by the CO_2_ inlet flow combined with the slow sweep rate of the OSA. This was confirmed by a series of experiments using different sweep rates for the OSA and conducting the experimental measurements with no gas flowing causing the slow sinusoidal variation to disappear. The test was performed several times on three different sensors with the same fabrication conditions and all showed a selective response to CO_2_ but with differing spectral sensitivities. Wavelength shifts ranged from 0.6 to 4 nm over the full range of CO_2_ concentrations, resulting in detection limits from 523 to 150 ppm at one atmosphere pressure. The detection limit was obtained by determining the wavelength shift as a function of CO_2_ concentration and using the spectral resolution of the resonance^37^. The differences in the resolution of each device can be attributed to small variations in the manual fabrication procedure used and other experimental and environmental parameters, such as matching the polarization of the illuminating light to the device, the central wavelength of the LSP resonances and fluctuations in temperature. The closest work in the literature to that reported here is reference 22, where CNTs were coated on a Bragg grating recorded in a 3.8 µ diameter fiber. Those authors were able to show that the Bragg wavelength changed when CO_2_ was introduced into the gas surrounding the fiber. Importantly, the authors did not demonstrate insensitivity to other gases as we have done here. Furthermore, a direct comparison of sensitivity and resolution between our work and reference 22 is not possible; the authors reported, but did not explain, why larger concentrations of CO_2_ resulted in smaller Bragg wavelength shifts.

The polarization dependence of the sensor was also investigated before and after the CNT coating was applied. It was found that the CNTs reduced the overall sensitivity of the optical strength to changing polarization. For example, rotating the azimuth of polarization from the optimum reduced the strength of the resonance by about 2.6 dB degree^−1^ before coating and only 1.8 dB degree^−1^ after the CNTs were added.

It is known that the shape of the supporting particles of the surface plasmons affects the polarization characteristics^38^. The polarization behavior described above suggests that the CNTs are supporting the plasmons or at least the plasmons are interacting with the CNTs.

## Conclusion

We demonstrate, what we believe to be the first chemically selective change in the optical response of CNTs to a specific molecule (CO_2_). This is distinctively different to previous indirect approaches, such as attaching fluorophores to the CNTs and using the CNTs as an effective quencher of the fluorescence^39^. In our case, the modification in the optical properties of the CNTs is observed using an optical fiber-based plasmonic sensing platform, which identifies a CO_2_-specific response of the sensing element with a sensitivity of Δ*λ*/Δ*n* ∼ −6200 nm RIU^−1^. This is the first time that direct monitoring of the optical properties of CNTs has been used as a mechanism for selective chemical sensing. Furthermore, this is the first demonstration of a new technique to monitor the physical characteristics of CNTs. This is also the first demonstration of species-specific optical fiber gas sensing that utilizes directly the optical properties of CNTs.

In addition, we have shown that the experimental results yield a practical approach to specific chemical detection and this is a significant step towards the realization of a practical gas sensor based upon the optical properties of CNTs. It is important to stress that CNTs can be functionalized to yield specific responses for various other chemicals^5,6,7,8,9,10,11^.

## Authors' contributions

TA and AR developed the original optical plasmonic gas sensor concept. TA modelled the behavior, designed and performed experiments and analyzed the data for the plasmonic devices. TA and RN fabricated the plasmonic devices. RA and AR developed and adhered the CNT coatings to the plasmonic devices. TA and KK designed and performed experiments for gas sensing. VK and TA characterized the devices. TA and KK developed the explanation for the sensor behavior. The manuscript was written by TA, RN, AR, KK, DJW, and PC. All authors discussed the results and commented on the manuscript. To access the data underlying this publication, please contact mailto:researchdata@aston.ac.uk., see http://dx.doi.org/10.17036/f70016d4-f2f3-46c1-91fd-e6902a92c8d0

## References

[bib1] Iijima S. Helical microtubules of graphitic carbon. Nature 1991; 354: 56–58.

[bib2] Bekyarova E, Sarkar S, Wang FH, Itkis ME, Kalinina I et al. Effect of covalent chemistry on the electronic structure and properties of carbon nanotubes and graphene. Acc Chem Res 2013; 46: 65–76. 23116475 10.1021/ar300177q

[bib3] Saito R, Hofmann M, Dresselhaus G, Jorio A, Dresselhaus MS. Raman spectroscopy of graphene and carbon nanotubes. Adv Phys 2011; 60: 413–550.

[bib4] Jorio A, Dresselhaus G, Dresselhaus MS. Carbon Nanotubes: Advanced Topics in the Synthesis, Structure, Properties and Applications. Berlin:Springer; 2008.

[bib5] Penzaa M, Rossi R, Alvisi M, Cassano G, Signore MA et al. Pt- and Pd-nanoclusters functionalized carbon nanotubes networked films for sub-ppm gas sensors. Sens Actuat B 2008; 135: 289–297.

[bib6] Zhang T, Mubeen S, Myung NV, Deshusses MA. Recent progress in carbon nanotube-based gas sensors. Nanotechnology 2008; 19: 332001. 21730614 10.1088/0957-4484/19/33/332001

[bib7] Mittal M, Kumar A. Carbon nanotube (CNT) gas sensors for emissions from fossil fuel burning. Sens Actuat B 2014; 203: 349–362.

[bib8] Sun GZ, Liu SW, Hua KF, Lv XY, Huang L et al. Electrochemical chlorine sensor with multi-walled carbon nanotubes as electrocatalysts. Electrochem Commun 2007; 9: 2436–2440.

[bib9] Bekyarova E, Davis M, Burch T, Itkis ME, Zhao B et al. Chemically functionalized single-walled carbon nanotubes as ammonia sensors. J Phys Chem B 2004; 108: 19717–19720.

[bib10] Zhang YM, Zhang DJ, Liu CB. Novel chemical sensor for cyanides: boron-doped carbon nanotubes. J Phys Chem B 2006; 110: 4671–4674. 16526700 10.1021/jp0602272

[bib11] Lu YJ, Partridge C, Meyyappan M, Li J. A carbon nanotube sensor array for sensitive gas discrimination using principal component analysis. J Electroanal Chem 2006; 593: 105–110.

[bib12] Wolfbeis OS. Fiber-optic chemical sensors and biosensors. Anal Chem 2008; 80: 4269–4283. 18462008 10.1021/ac800473b

[bib13] Allsop T, Neal R, Mou C, Brown P, Saied S et al. Exploitation of multilayer coatings for infrared surface plasmon resonance fiber sensors. Appl Opt 2009; 48: 276–286. 19137038 10.1364/ao.48.000276

[bib14] Raether H. Surface Plasmons on Smooth and Rough Surfaces and on Gratings. New York: Academic; 1997.

[bib15] Allsop T, Nagel D, Neal R, Davies EM, Mou C et al. Aptamer-based surface plasmon fibre sensor for thrombin detection. Proc SPIE 2010; 7715: 77151C.

[bib16] Chopra S, McGuire K, Gothard N, Rao AM, Pham A. Selective gas detection using a carbon nanotube sensor. Appl Phys Lett 2003; 83: 2280–2282.

[bib17] Rahimi M, Singh JK, Babu DJ, Schneider JJ, Müller-Plathe F. Understanding carbon dioxide adsorption in carbon nanotube arrays: molecular simulation and adsorption measurements. J Phys Chem C 2013; 117: 13492–13501.

[bib18] Cinke M, Li J, Bauschlicher Jr CW, Ricca A, Meyyappan M. CO_2_ adsorption in single-walled carbon nanotubes. Chem Phys Lett 2003; 376: 761–766.

[bib19] Kumari GS, Rao JVR. Detection of NH_3_ & CO_2_ using arbon nanotubes at room temperature. Int J Nanotechnol Appl 2013; 3: 11–18.

[bib20] Heiblum M, Harris JH. Analysis of curved optical waveguides by conformal transformation. IEEE J Quantum Electron 1975; QE-11: 75–83.

[bib21] Williams DL, Davey ST, Kashyap R, Armitage JR, Ainslie BJ. UV spectroscopy of optical fibers and preforms. Proc SPIE 1991; 1516: 29–37.

[bib22] Allsop TDP, Neal R, Mou C, Kalli K, Saied S et al. Formation and characterization of ultra-sensitive surface plasmon resonance sensor based upon a nano-scale corrugated multi-layered coated D-shaped optical fiber. IEEE J Quantum Electron 2012; 48: 394–405.

[bib23] Bronstein AM, Kimmel R. Numerical Geometry of Non-rigid Shapes. Berlin: Springer Science & Business Media; 2008.

[bib24] Giordani S, Bergin SD, Nicolosi V, Lebedkin S, Kappes MM et al. Debundling of single-walled nanotubes by dilution: observation of large populations of individual nanotubes in amide solvent dispersions. J Phys Chem B 2006; 110: 15708–15718. 16898715 10.1021/jp0626216

[bib25] Hasan T, Scardaci V. Stabilization and “debundling” of single-wall carbon nanotube dispersions in N-methyl-2-pyrrolidone (NMP) by polyvinylpyrrolidone (PVP). J Phys Chem C 2007; 111: 12594–12602.

[bib26] Haes AJ, van Duyne RP. A unified view of propagating and localized surface plasmon resonance biosensors. Anal Bioanal Chem 2004; 379: 920–930. 15338088 10.1007/s00216-004-2708-9

[bib27] Verma RK, Gupta BD. Surface plasmon resonance based fiber optic sensor for the IR region using a conducting metal oxide film. J Opt Soc Am A 2010; 27: 846–851. 10.1364/JOSAA.27.000846 20360826

[bib28] Kim YC, Peng W, Banerji S, Booksh KS. Tapered fiber optic surface plasmon resonance sensor for analyses of vapor and liquid phases. Opt Lett 2005; 30: 2218–2220. 16190423 10.1364/ol.30.002218

[bib29] Xiao GZ, Adnet A, Zhang ZY, Sun FG, Grover CP. Monitoring changes in the refractive index of gases by means of a fiber optic Fabry-Perot interferometer sensor. Sens Actuat A 2005; 118: 177–182.

[bib30] Monzón-Hernández D, Luna-Moreno D, Martínez-Escobar D. Fast response fiber optic hydrogen sensor based on palladium and gold nano-layers. Sens Actuat B 2009; 136: 562–566.

[bib31] Allsop T, Neal R, Chengbo M, Kalli K, Webb D. Highly sensitive, localized surface plasmon resonance fiber device for environmental sensing, based upon a structured bi-metal array of nano-wires. Opt Lett 2014; 39: 5798–5801. 25361088 10.1364/OL.39.005798

[bib32] Shivananju BN, Yamdagni S, Fazuldeen R, Sarin Kumar AK, Hegde GM et al. CO_2_ sensing at room temperature using carbon nanotubes coated core fiber Bragg grating. Rev Sci Instrum 2013; 84: 065002. 23822371 10.1063/1.4810016

[bib33] Chistiakova MV, Armani AM. Optical detection of CO and CO_2_ temperature dependent desorption from carbon nanotube clusters. Nanotechnology 2014; 25: 395201. 25189292 10.1088/0957-4484/25/39/395201

[bib34] Sinha N, Ma J, Yeow JTW. Carbon nanotubes-based sensors. J Nanosci Nanotechnol 2006; 6: 573–590. 16573108 10.1166/jnn.2006.121

[bib35] Modi A, Koratkar N, Lass E, Wei BQ, Ajayan PM. Miniaturized gas ionization sensors using carbon nanotubes. Nature 2003; 424: 171–174. 12853951 10.1038/nature01777

[bib36] Liu WW, Azizan A, Chai SP, Mohamed AR, Tye CT. Optimisation of reaction conditions for the synthesis of single‐walled carbon nanotubes using response surface methodology. Can J Chem Eng 2012; 90: 489–505.

[bib37] Hu JJ, Sun XC, Agarwal A, Kimerling LC. Design guidelines for optical resonator biochemical sensors. J Opt Soc Am B 2009; 26: 1032–1041.

[bib38] Miller MM, Lazarides AA. Sensitivity of metal nanoparticle surface plasmon resonance to the dielectric environment. J Phys Chem B 2005; 109: 21556–21565. 16853799 10.1021/jp054227y

[bib39] Li C, Shi GQ. Carbon nanotube-based fluorescence sensors. J Photochem Photobiol C 2014; 19: 20–34.

